# Teaching the Deaf and Dumb

**Published:** 1889-03-02

**Authors:** 


### TEACHING THE DEAF AND DUMB.

Mr. William Van Praagh, director of the school and training college for teachers of the Association for the Oral Instruction of the Deaf and Dumb, has published a series of papers on subjects relating to the above. Dumbness, the author asserts, is caused by deafness, and deafness may be either congenital or acquired. At present there are three systems of teaching the deaf and dumb; the oral plan, which teaches by articulation and lip-reading; the sign system, which employs artificial signs and the manual alphabet; thirdly, a combination of both methods. But the great principle is to find a substitute for the faculty of hearing in the sight. Every sound we articulate produces vibration of the face and throat, as well as of the lips. The deaf can be made to see what is said by carefully watching these movements, and the sight thus becomes the substitute for hearing. Similarly, the deaf person is taught speech by imitation. But to teach the deaf and dumb properly requires the utmost energy and devotion, In Mr. Van Praagh's opinion, the best manner of teaching a deaf child is to discard all kind of signs for the purely oral system, and at least an eight years' course of instruction is required. Deaf boys and girls, once able to express themselves and to follow what is said by others, are almost on an equality with persons in possession of the ordinary faculties. It is not everyone, however, who can be a deaf and dumb teacher. A long training is necessary for the teacher before he can successfully impart the secret of "hearing with the eyes" to deaf mutes.

